# Supplementary material for: Phylogenetic Diversity of Ossification Patterns in the Avian Vertebral Column: A Review and New Data from the Domestic Pigeon and Two Species of Grebes
Source: Biology (Basel). 2022 Jan 24;11(2):180. doi: 10.3390/biology11020180 (PMC8869719; doi:10.3390/biology11020180)
Supplement: Supplementary file 1 [file biology-11-00180-s001.zip › Table S1.pdf]

Table S1. Estimated divergence dates for given clades of the avian phylogenetic tree for the temporally-calibrated ancestral state reconstructions.

**Clade**	**Divergence date**	**Source**
Eudromia + *Dromaius*	45	Kuhl et al. [38]
Apteryx + *Dromaius*	50	Kuhl et al. [38]
Rhea + *Dromaius*	51	Kuhl et al. [38]
Struthio + *Dromaius*	58	Kuhl et al. [38]
Coturnix + *Synoicus*	18	TimeTree [40]
Coturnix + *Gallus*	20	Prum et al. [37]
Gallus + *Meleagris*	32	TimeTree [40]
Anas + *Somateria*	20	TimeTree [40]
Anas + *Cairina*	23	TimeTree [40]
Larus + *Chroicocephalus*	6	TimeTree [40]
Larus + *Sterna*	16	Kuhl et al. [38]
Larus + *Stercorarius*	22	Kuhl et al. [38]
Corvus + *Taeniopygia*	34	Kuhl et al. [38]
Corvus + *Myiopsitta*	62	Kuhl et al. [38]
Neoaves	69	Kuhl et al. [38]
Galloanserae	63	Kuhl et al. [38]
Neognathae	81	Kuhl et al. [38]
Neornithes	94	Kuhl et al. [38]
