# Supplementary material for: Phylogenetic Diversity of Ossification Patterns in the Avian Vertebral Column: A Review and New Data from the Domestic Pigeon and Two Species of Grebes
Source: Biology (Basel). 2022 Jan 24;11(2):180. doi: 10.3390/biology11020180 (PMC8869719; doi:10.3390/biology11020180)
Supplement: Supplementary file 1 [file biology-11-00180-s001.zip › Table S2.pdf]

Table S2. Results of ancestral state reconstructions for major avian clades, represented by at least three species in our dataset. The values reported in brackets in maximum likelihood analyses are proportional likelihoods. Abbreviations: CL – cervical locus, TL – thoracic locus.

**Clade**	**Maximum parsimony**	**Maximum likelihood (equal length branches)**	**Maximum likelihood (time-calibrated branch lengths)**
Neornithes	CL: present<br>TL: present	CL: present (1)<br>TL: likely present (0.906)	CL: present (1)<br>TL: likely present (0.788)
Palaeognathae	CL: present<br>TL: present	CL: present (1)<br>TL: likely present (0.981)	CL: present (1)<br>TL: likely present (0.950)
Neognathae	CL: present<br>TL: present	CL: present (1)<br>TL: likely present (0.872)	CL: present (1)<br>TL: likely present (0.760)
Galloanserae	CL: present<br>TL: ambiguous	CL: present (1)<br>TL: ambiguous but likely present (0.558)	CL: present (1)<br>TL: likely present (0.666)
Galliformes	CL: present<br>TL: ambiguous	CL: present (1)<br>TL: ambiguous but likely present (0.523)	CL: present (1)<br>TL: likely present (0.732)
Gallus + *Coturnix*	CL: present<br>TL: present	CL: present (1)<br>TL: likely present (0.924)	CL: present (1)<br>TL: likely present (0.983)
Anseriformes	CL: present<br>TL: absent	CL: present (1)<br>TL: likely absent (0.710)	CL: present (1)<br>TL: likely absent (0.732)
Neoaves	CL: present<br>TL: present	CL: present (1)<br>TL: likely present (0.979)	CL: present (1)<br>TL: likely present (0.759)
Charadriiformes + Psittacopasserae	CL: present<br>TL: present	CL: present (1)<br>TL: likely present (0.849)	CL: present (1)<br>TL: likely present (0.748)
Charadriiformes	CL: present<br>TL: present	CL: present (1)<br>TL: likely present (0.972)	CL: present (1)<br>TL: likely present (0.989)
Psittacopasserae	CL: present<br>TL: absent	CL: present (1)<br>TL: likely absent (0.869)	CL: present (1)<br>TL: ambiguous but likely present (0.560)
